# An immune-related lncRNA model for predicting prognosis, immune landscape and chemotherapeutic response in bladder cancer

**DOI:** 10.1038/s41598-022-07334-w

**Published:** 2022-02-25

**Authors:** Jian Hou, Songwu Liang, Zhimin Xie, Genyi Qu, Yong Xu, Guang Yang, Cheng Tang

**Affiliations:** 1 Department of Urology, Zhuzhou Central Hospital, Zhuzhou, 412007 China; 2 Division of Urology, Department of Surgery, The University of Hongkong-Shenzhen Hospital, Shenzhen, 518000 China

**Keywords:** Bladder, Bioinformatics

## Abstract

Long noncoding RNAs (lncRNAs) participate in cancer immunity. We characterized the clinical significance of an immune-related lncRNA model and evaluated its association with immune infiltrations and chemosensitivity in bladder cancer. Transcriptome data of bladder cancer specimens were employed from The Cancer Genome Atlas. Dysregulated immune-related lncRNAs were screened via Pearson correlation and differential expression analyses, followed by recognition of lncRNA pairs. Then, a LASSO regression model was constructed, and receiver operator characteristic curves of one-, three- and five-year survival were established. Akaike information criterion (AIC) value of one-year survival was determined as the cutoff of high- and low-risk subgroups. The differences in survival, clinical features, immune cell infiltrations and chemosensitivity were compared between subgroups. Totally, 90 immune-related lncRNA pairs were identified, 15 of which were screened for constructing the prognostic model. The area under the curves of one-, three- and five-year survival were 0.806, 0.825 and 0.828, confirming the favorable predictive performance of this model. According to the AIC value, we clustered patients into high- and low-risk subgroups. High-risk score indicated unfavorable outcomes. The risk model was related to survival status, age, stage and TNM. Compared with conventional clinicopathological characteristics, the risk model displayed higher predictive efficacy and served as an independent predictor. Also, it could well characterize immune cell infiltration landscape and predict immune checkpoint expression and sensitivity to cisplatin and methotrexate. Collectively, the model conducted by paring immune-related lncRNAs regardless of expressions exhibits a favorable efficacy in predicting prognosis, immune landscape and chemotherapeutic response in bladder cancer.

## Introduction

Bladder cancer is responsible for almost 170,000 deaths globally each year, mainly including two subtypes: non-muscle invasive (75%) and muscle invasive (25%)^1^. At present, cystoscopy represents the gold standard of clinical tools for diagnosing bladder cancer. Nevertheless, this procedure exhibits high invasiveness, and there is the consequence of false-negatives sporadically occurring due to the difficulty in detecting carcinoma in situ^2^. Despite much progress in therapeutic strategies such as tumor resection, chemotherapy, and radiotherapy, survival duration and therapeutic responses vary among subjects. Due to high mutational burden, immune checkpoint inhibitors (ICIs) have been approved in advanced bladder cancer^3^. However, the overall response rate is merely 15–25%^4^, which highlights the importance of discovering biomarkers that may be predictive of treatment responses. As a highly heterogeneous malignancy, the etiology and clinicopathological manifestations vary among individuals. Growing evidence suggests that immunity is related to survival and therapeutic effects of bladder cancer^5^. For instance, targeting myeloid-derived suppressor cells (MDSCs) may heighten the therapeutic effects of ICIs for cisplatin-resistant bladder cancer^6^. Tumor‑infiltrating M2 macrophages are related to undesirable overall and disease‑specific survival duration^7^. Hence, screening reliable immune-related prognostic indicators is of importance for bladder cancer^8^.

Extensive RNA sequencing (RNA-seq) profiles by The Cancer Genome Atlas (TCGA) have suggested the implications of epigenetic, transcriptional, and post-transcriptional regulation of long noncoding RNAs (lncRNAs) in diagnosing and curing bladder cancer^9–11^. LncRNAs display higher specificity to biological states compared to coding RNAs^12^. Molecular characterizations have motivated to optimize actionable therapeutic strategies against bladder cancer^13^. As confirmed, lncRNAs mediate innate and adaptive immunity of bladder cancer through the functional states of immune cells and relevant pathways and genes^14^. For instance, lncRNA MIR4435-2HG contributes to unfavorable prognoses as well as high immune infiltrations in bladder cancer^15^. Recently, immune-related lncRNA signatures have been conducted for evaluating prognoses and immune infiltrations of bladder cancer^16–18^. Hence, this study attempted to develop a risk model constructed by immune-related lncRNA pairs for predicting the survival outcomes by modeling algorithms, paring, and iterations, immunotherapy, and chemotherapy of bladder cancer patients.

## Results

### Identifying dysregulated immune-related lncRNAs in bladder cancer

**Figure 1:**
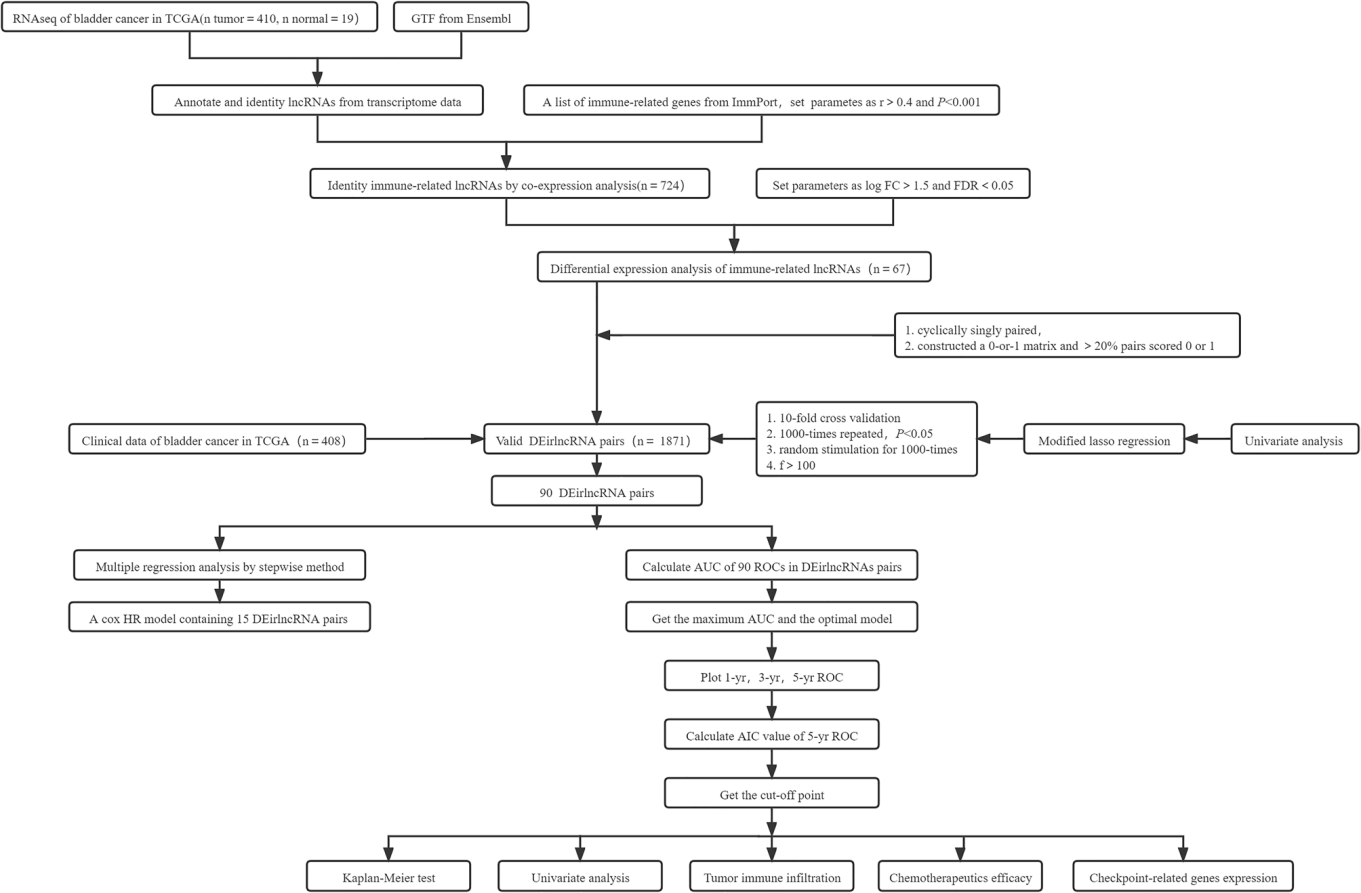
The workflow of this study.

**Figure 2:**
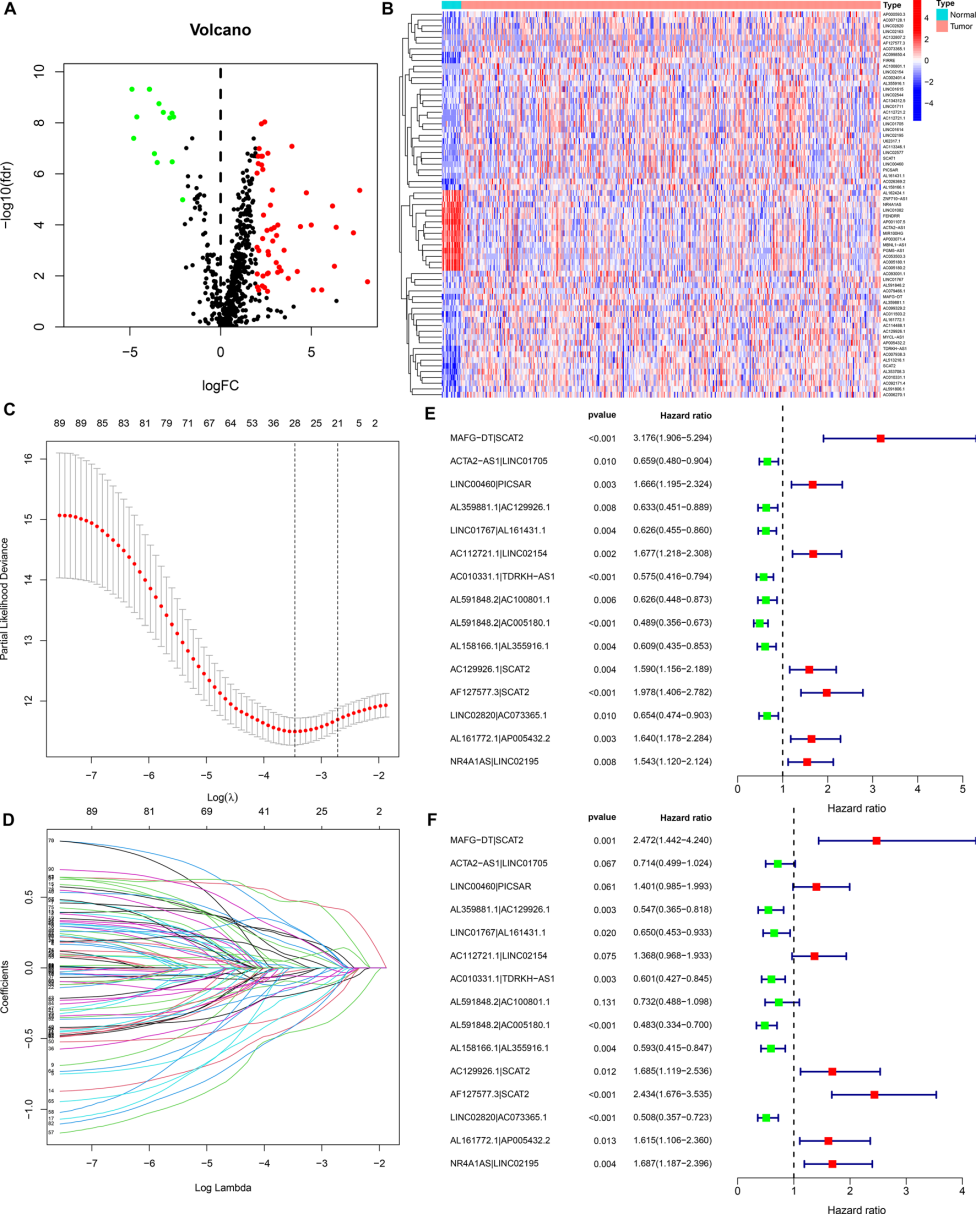
Developing a prognostic immune-related lncRNA signature for bladder cancer. (**A**) Volcano diagram of immune-related lncRNAs that displayed abnormal expression in bladder cancer and normal tissue specimens. Red dots: up-regulation and green dots: down-regulation. (**B**) Hierarchical clustering analyses of the dysregulated expression patterns of these immune-related lncRNAs between bladder cancer and normal tissue specimens. Red: up-regulation and blue: down-regulation. (**C**) Elucidating LASSO coefficient profiling of these prognostic lncRNAs. (**D**) Validating tuning parameter selection for LASSO regression model. (**E**) Univariate cox regression analyses of the dysregulated immune-related lncRNAs that may significantly impact bladder cancer’s survival. Red: risk factor and green: protective factor. (**F**) Multivariate Cox regression analyses of the candidate prognostic lncRNAs.

Figure 1 depicted the workflow of this study. Here, transcriptome profiles of bladder cancer and normal specimens were obtained from TCGA and the lncRNAs were extracted. Immune-related lncRNAs that were distinctly correlated to immune-related genes were selected according to correlation coefficient > 0.4 and p < 0.001. As a result, 724 immune-related lncRNAs were identified (Supplementary table 1). Their expressions were compared between bladder cancer and normal specimens. Our data showed that 14 immune-related lncRNAs displayed down-regulation while 53 exhibited up-regulation in bladder cancer compared to normal specimens (Fig. 2A, B and Supplementary table 2).

### Developing dysregulated immune-related lncRNA pairs and a risk model in bladder cancer

Utilizing an iteration loop and 0-or-1 matrices, 1871 dysregulated immune-related lncRNA pairs were recognized (Supplementary table 3). As depicted in univariate cox regression analyses, 90 lncRNA pairs could significantly impact bladder cancer subjects’ survival (Supplementary table 4). Above pairs were screened through a LASSO model. As a result, 15 dysregulated immune-related lncRNA pairs including MAFG-DT|SCAT2, ACTA2-AS1|LINC01705, LINC00460|PICSAR, AL359881.1|AC129926.1, LINC01767|AL161431.1, AC112721.1|LINC02154, AC010331.1|TDRKH-AS1, AL591848.2|AC100801.1, AL591848.2|AC005180.1, AL158166.1|AL355916.1, AC129926.1|SCAT2, AF127577.3|SCAT2, LINC02820|AC073365.1, AL161772.1|AP005432.2, and NR4A1AS|LINC02195 were put into this risk model (Fig. 2C, D). Through uni- and multivariate cox regression analyses, these pairs displayed distinct associations with survival outcomes (Fig. 2E, F). According to the coefficients and expressions of lncRNA pairs (Table 1), RS was calculated for bladder cancer subjects.

**Table 1:** Regression coefficients of each factor in this prognostic immune-related lncRNA signature.

LncRNAs	Coefficient	HR	HR.95L	HR.95H	P-value
MAFG-DT|SCAT2	0.9052	2.4725	1.4417	4.2397	0.0010
ACTA2-AS1|LINC01705	− 0.3363	0.7144	0.4986	1.0235	0.0668
LINC00460|PICSAR	0.3370	1.4007	0.9846	1.9928	0.0610
AL359881.1|AC129926.1	− 0.6039	0.5467	0.3653	0.8179	0.0033
LINC01767|AL161431.1	− 0.4304	0.6503	0.4532	0.9331	0.0195
AC112721.1|LINC02154	0.3135	1.3682	0.9684	1.9331	0.0754
AC010331.1|TDRKH-AS1	− 0.5091	0.6010	0.4273	0.8454	0.0034
AL591848.2|AC100801.1	− 0.3116	0.7323	0.4885	1.0977	0.1314
AL591848.2|AC005180.1	− 0.7269	0.4834	0.3339	0.6999	0.0001
AL158166.1|AL355916.1	− 0.5227	0.5929	0.4152	0.8468	0.0040
AC129926.1|SCAT2	0.5215	1.6846	1.1189	2.5364	0.0125
AF127577.3|SCAT2	0.8895	2.4339	1.6760	3.5346	2.97E−06
LINC02820|AC073365.1	− 0.6777	0.5078	0.3566	0.7231	0.0002
AL161772.1|AP005432.2	0.4795	1.6153	1.1057	2.3599	0.0132
NR4A1AS|LINC02195	0.5227	1.6866	1.1873	2.3960	0.0035

*HR* hazard ratio, *HR.95L* 95% CI lower limit, *HR.95H* 95% CI upper limit.

### Evaluating the predictive performance of this risk model for prognoses

**Figure 3:**
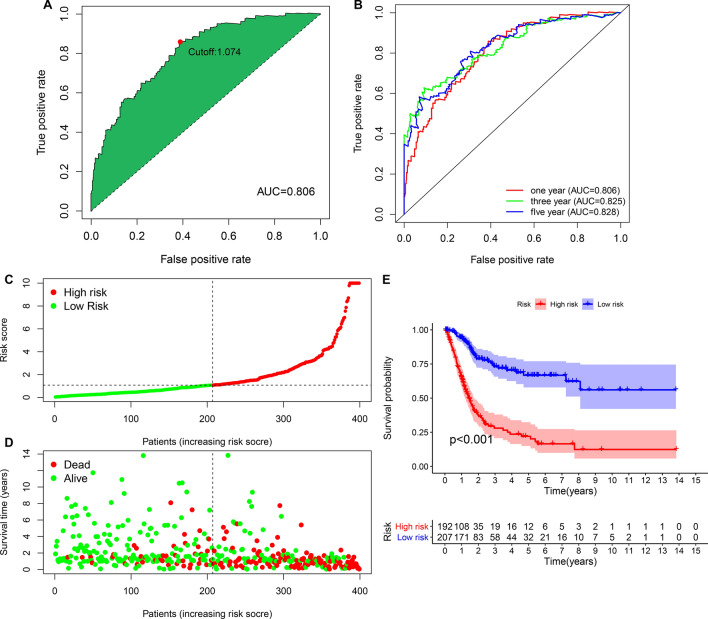
Evaluating the predictive performance of this immune-related lncRNA signature on bladder cancer prognoses. (**A**) ROC curves of the immune-related lncRNA signature for bladder cancer subjects. The maximum inflection point was the cut-off point that was calculated with the AIC method. The AUC value was calculated for evaluating the predictive efficacy of this signature in bladder cancer prognoses. (**B**) The one-, three- and five-year ROC curves of this signature. (**C**) Calculating the risk score of each bladder cancer subject and distinguishing patients into high and low risk subgroups based on the cutoff value (vertical dotted line). Red: high risk and green: low risk. (**D**) Visualizing the distribution of survival status in high and low risk bladder cancer subjects. Red dots: alive and green dots: alive. Vertical dotted line represented the cutoff value of two subgroups. (**E**) Kaplan–Meier curves of overall survival between high and low risk patients.

The cutoff value that differentiated bladder cancer subjects into high- and low-risk subgroups was 1.074 according to the AIC of one-year survival (Fig. 3A). The AUC of one-year survival was 0.806. This indicated the favorable predictive efficacy of this risk model. Furthermore, we conducted the ROCs of three- and five-year survival. The AUCs of three- and five-year survival were 0.825 and 0.828, demonstrating that this risk model was also utilized for predicting three- and five-year clinical outcomes of bladder cancer (Fig. 3B). According to the cutoff value, we clustered patients into high- and low-risk subgroups (Fig. 3C). The distributions of survival status between subgroups were depicted in Fig. 3D. High-risk subgroup possessed more dead patients in comparison to low-risk subgroup. The differences in survival duration were compared between subgroups. In Fig. 3E, low-risk patients were predictive of favorable clinical outcomes compared to high-risk patients (p < 0.001).

### Associations between clinical features and this risk model

**Figure 4:**
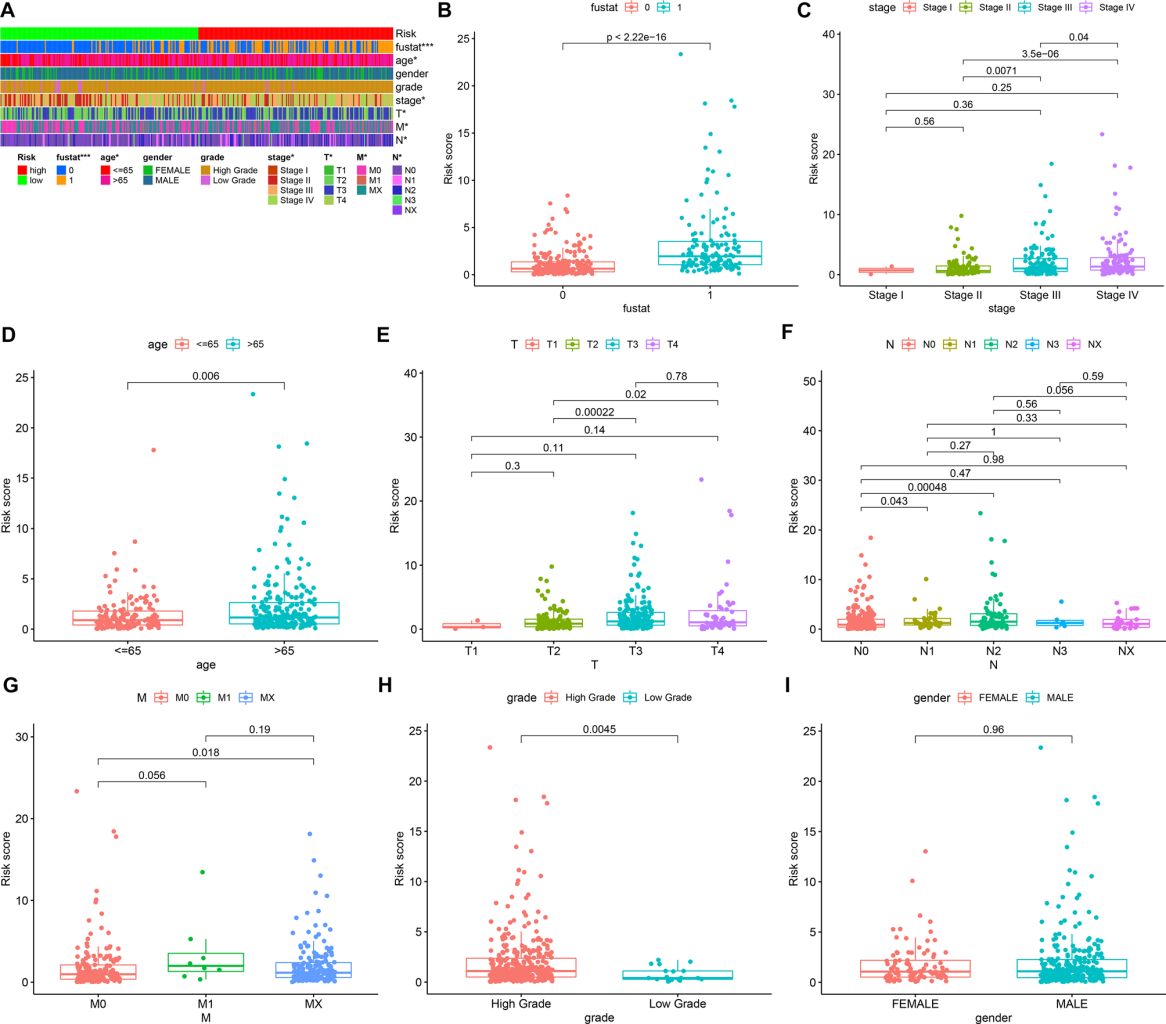
Associations between this prognostic immune-related lncRNA signature and clinicopathological characteristics of bladder cancer. (**A**) Heatmaps of the visualization of clinicopathological characteristics: survival status, age, gender, grade, stage, T, N and M in high and low bladder cancer subjects. *p < 0.05; ***p < 0.001. Comparing the risk score in different clinicopathological characteristics: (**B**) survival status (0: dead; 1: alive), (**C**) stage I–IV, (**D**) age ≤ 65 and > 65, (**E**) T1–4, (**F**) N0-X, (**G**) M0-X, (**H**) grade and (**I**) gender.

Figure 4A depicted the associations between clinical features and this risk model in bladder cancer. We found that the risk model was in relation to survival status (p < 0.001), age (p < 0.05), stage (p < 0.05), T (p < 0.05), N (p < 0.05) and M (p < 0.05) of bladder cancer patients. The differences in RS were compared among different subgroups of clinical features. As shown in our data, patients with dead status exhibited higher RS than those with alive status (p < 2.22e−16; Fig. 4B). In Fig. 4C, patients in stage III-IV had elevated RS compared to those with stage I-II. Furthermore, > 65 patients displayed increased RS than ≤ 65 subjects (p = 0.006; Fig. 4D). As depicted in Fig. 4E, subjects with T3–4 possessed higher RS than those with T1–2. Compared to patients with N0, increased RS was detected in those with N1–2 (Fig. 4F). Also, higher RS was found in patients with M1 or Mx than M0 (Fig. 4G). There was elevated RS in high grade than low grade specimens (p = 0.0045; Fig. 4H). Nevertheless, no significant difference in RS was found between female and male specimens (Fig. 4I). Hence, this risk model might be relation to bladder cancer progression and metastases.

### This risk model as an independent prognostic predictor

**Figure 5:**
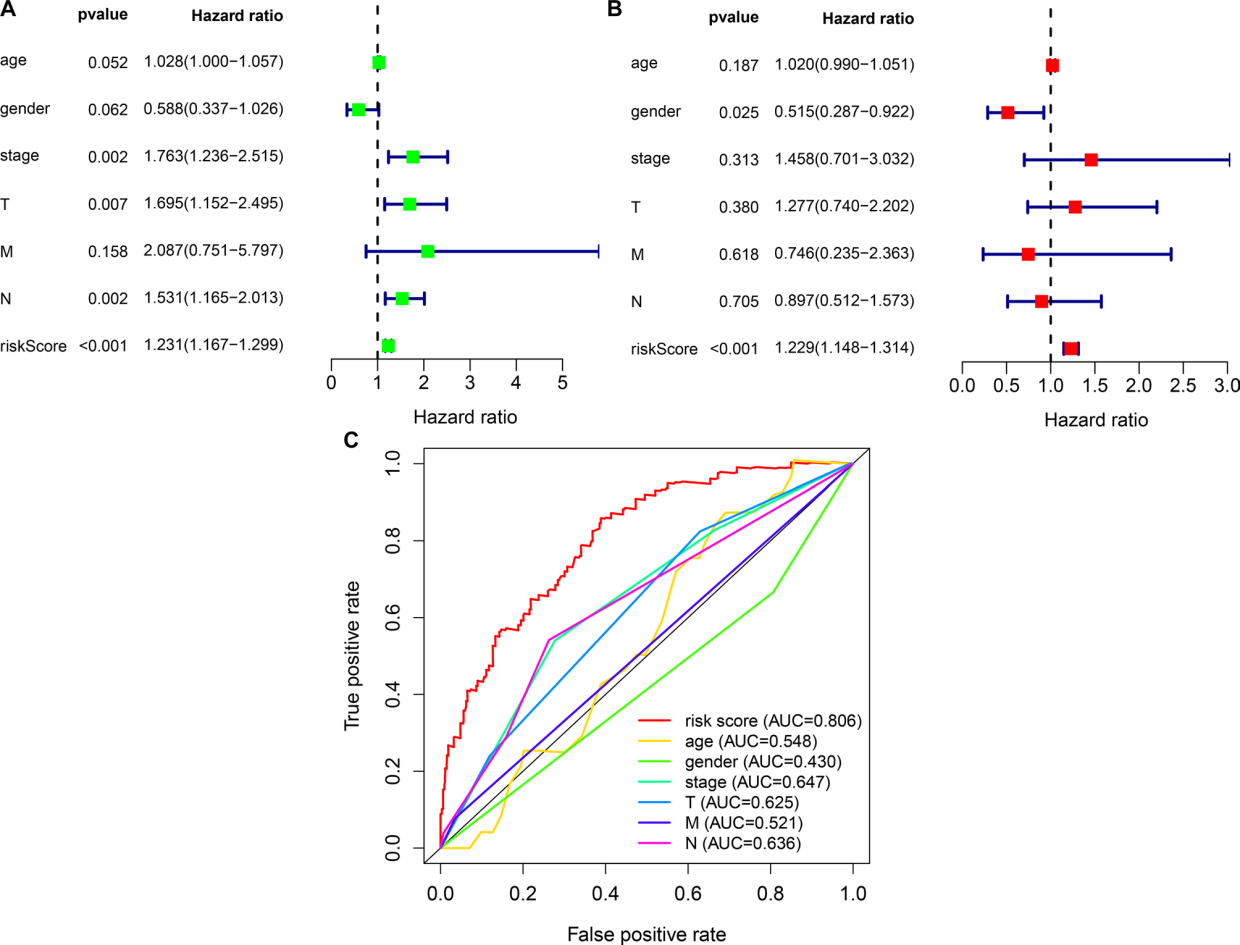
Assessing the predictive independency of this prognostic immune-related lncRNA signature for bladder cancer prognoses. (**A**) Univariate cox regression analyses of the correlations of age, gender, stage, T, N, M and risk score with bladder cancer prognoses. (**B**) Multivariate cox regression for evaluating the independent predictive factors. (**C**) Comparing the AUC values of age, gender, stage, T, N, M and risk score.

As depicted in univariate cox regression analyses, stage, T, N, and risk model were in relation to bladder cancer prognoses (Fig. 5A). This was indicative that above factors might impact patients’ clinical outcomes. To evaluate the predictive independency, multivariate cox regression analyses were conducted. In Fig. 5B, this risk model might be independently predictive of patients’ prognoses. ROCs were conducted for comparing their differences in predictive performance of one-year survival. We found that this risk model possessed the highest AUC value (Fig. 5C), demonstrating the favorable efficacy in predicting prognoses.

### This risk model might predict immune cell landscape of bladder cancer

**Figure 6:**
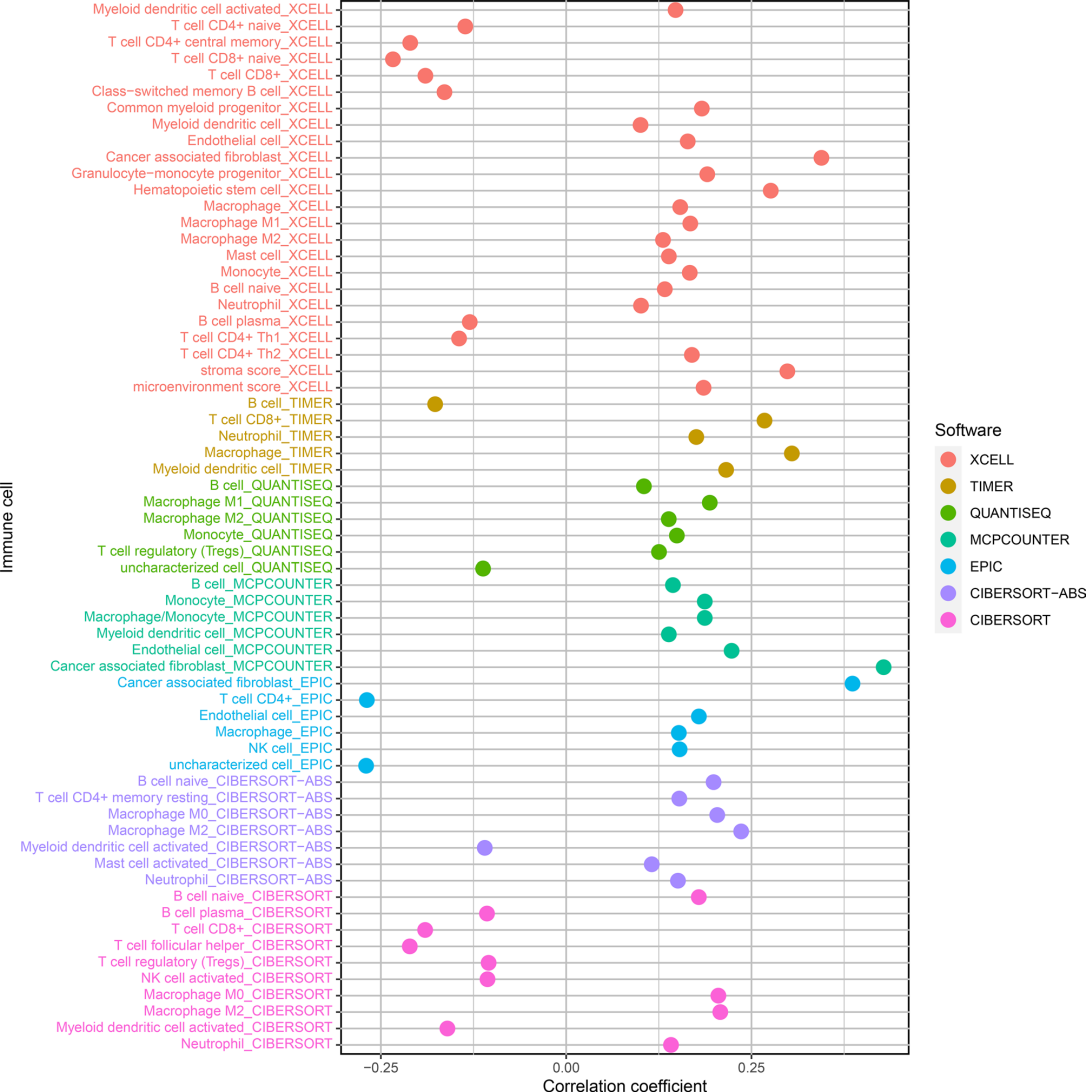
Correlations between risk score and immune cell infiltrations of bladder cancer specimens by following software: XCELL; TIMER; QUANTISEQ; MCPCOUNTER; EPIC; CIBERSORT-ABS and CIBERSORT.

This study estimated immune cell infiltrations of bladder cancer specimens through XCELL, TIMER, QUANTISEQ, MCPCOUNTER, EPIC, CIBERSORT-ABS and CIBERSORT algorithms. Correlations between risk model and immune cell infiltrations were estimated via Spearson correlation test, as depicted in Fig. 6. Our data demonstrated that high-risk specimens possessed increased infiltrations of myeloid dendritic cell, B cell native, macrophage M0 and M2, neutrophil and T cell CD8 (Supplementary Fig. 1).

### Assessment of immune checkpoints with this risk model

**Figure 7:**
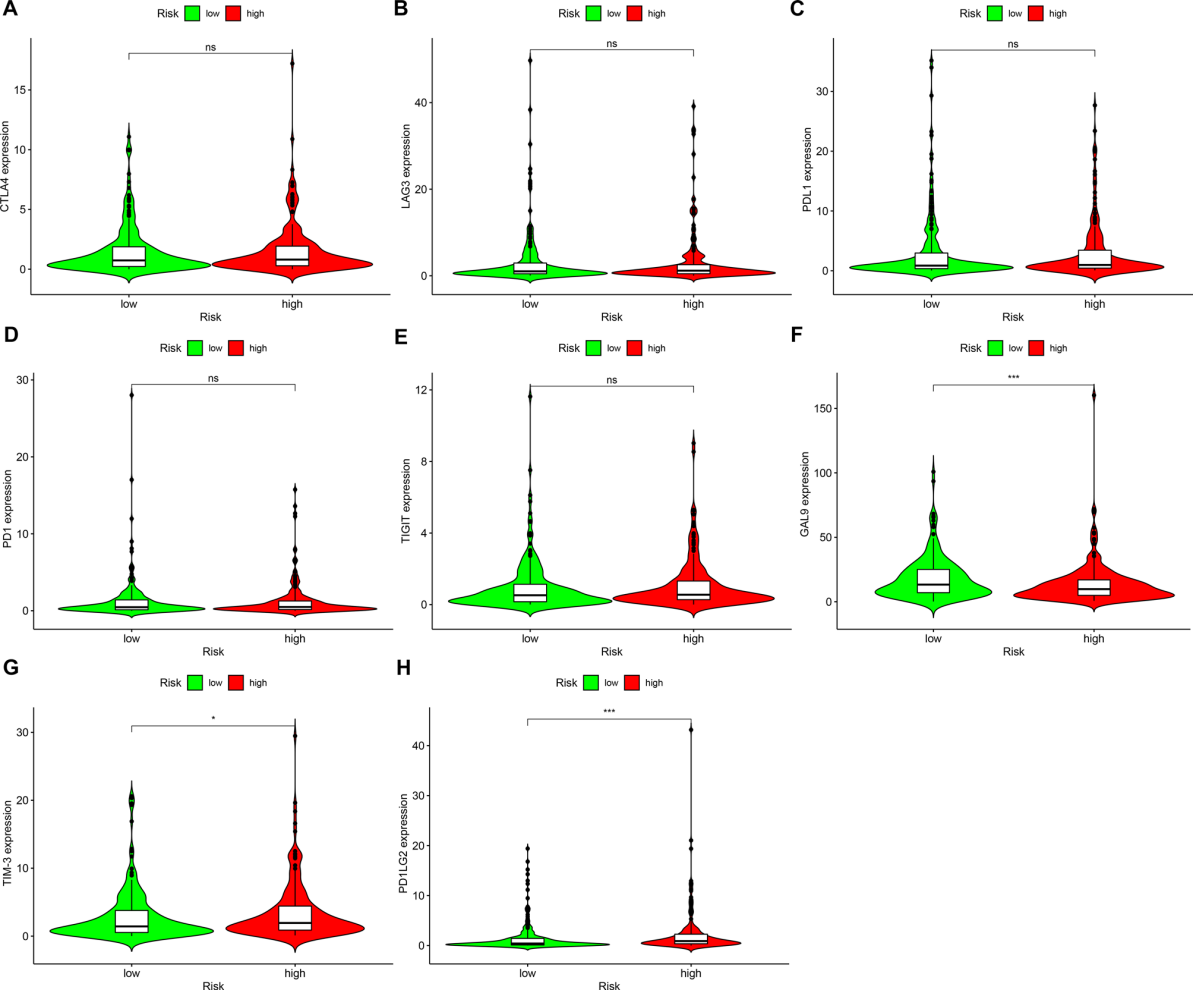
Correlations between risk model and immune checkpoints in bladder cancer. Comparing the expressions of (**A**) CTLA4; (**B**) LAG3; (**C**) PDL1; (**D**) PD1; (**E**) TIGIT; (**F**) GAL9; (**G**) TIM-3 and (**H**) PD1LG2 in high and low bladder cancer subjects. Ns: not significant; *p < 0.05; ***p < 0.001.

Currently, ICIs have been approved for bladder cancer treatment. Hence, this study observed the correlations between this risk model and immune checkpoints in bladder cancer specimens. No significant differences in CTLA4 (Fig. 7A), LAG3 (Fig. 7B), PLD1 (Fig. 7C), PD1 (Fig. 7D) and TIGIT (Fig. 7E) expressions were detected between high- and low-risk specimens. Nevertheless, GAL9 displayed elevated expression in low- than high-risk specimens (Fig. 7F; p < 0.001). Inversely, higher TIM-3 (Fig. 7G; p < 0.05) and PD1LG2 (Fig. 7H; p < 0.001) expressions were found in high-risk specimens in comparison to low-risk specimens.

### Analysis of the associations between this risk model and chemosensitivity

**Figure 8:**
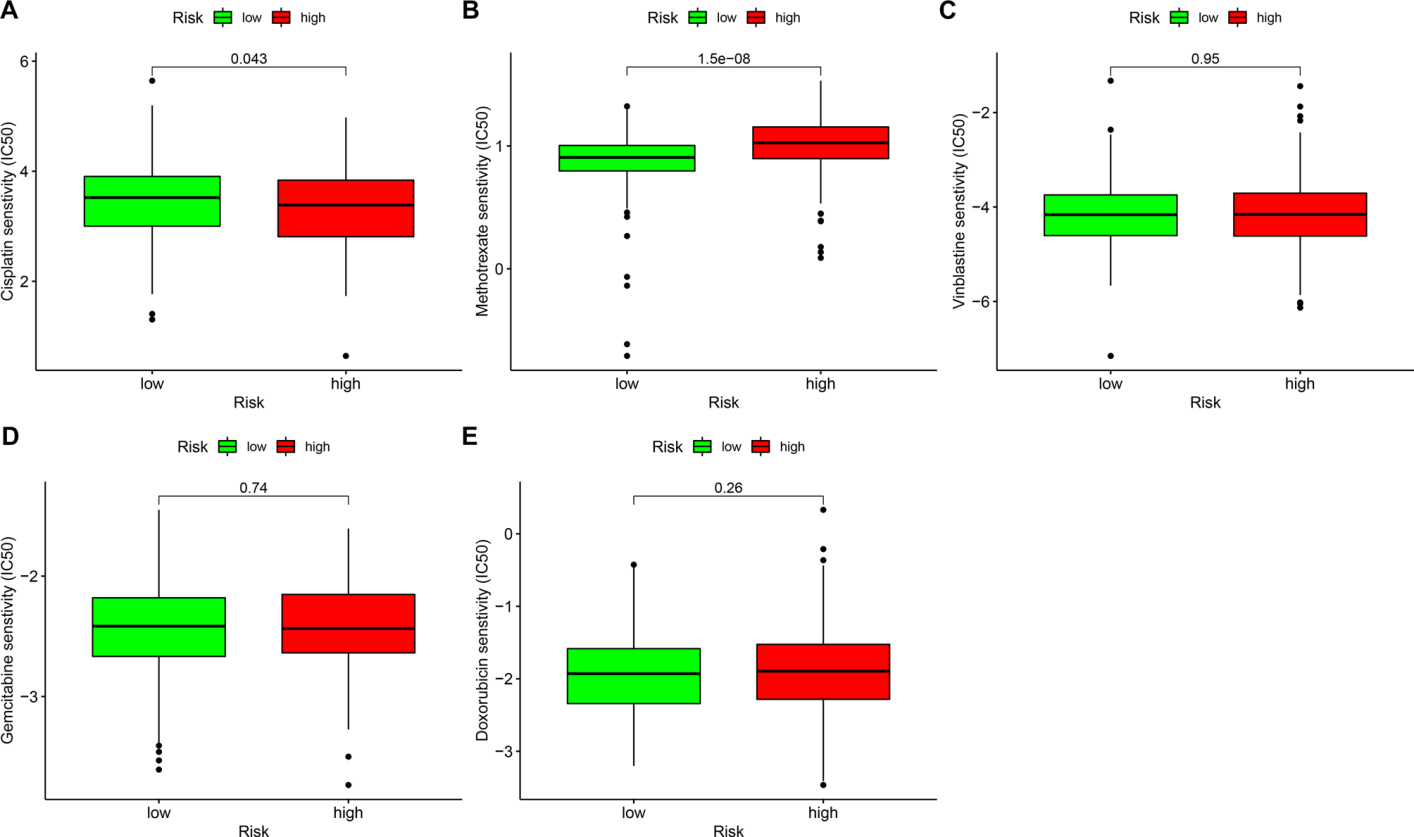
Correlations between risk model and the sensitivity to chemotherapy drugs in bladder cancer. Comparing the IC50 values of (**A**) cisplatin; (**B**) methotrexate; (**C**) vinblastine; (**D**) gemcitabine and (**E**) doxorubicin in high and low bladder cancer subjects.

The associations between this risk model and chemosensitivity were evaluated in bladder cancer specimens. Our data showed that high-risk patients exhibited decreased IC50 values of cisplatin in comparison to low-risk subjects (Fig. 8A; p = 0.043). This indicated that high-risk scores were predictive of higher sensitivity to cisplatin. In Fig. 8B, reduced IC50 values of methotrexate were found in low-risk specimens than high-risk specimens (p = 1.5e−08), demonstrating that low-risk scores were in relation to higher sensitivity to methotrexate. Furthermore, this study evaluated the differences in IC50 values of vinblastine (Fig. 8C), gemcitabine (Fig. 8D) and doxorubicin (Fig. 8E) between high- and low-risk specimens. Nevertheless, no significant differences were found.

## Discussion

LncRNAs have been confirmed to be in relation to cancer immunity and tumor microenvironment in bladder cancer^19^. Several immune-related lncRNA models have been constructed according to published literature^16,20,21^. Nevertheless, these signature models are developed on the basis of expression quantifications of immune-related lncRNAs. Herein, this study recognized the immune-related lncRNA pairs and constructed a reliable and independent risk model through combining two lncRNAs, not adopting their expression levels^22^.

Here, we firstly screened immune-related lncRNAs by Pearson correlation analyses. Different from previous research, dysregulated immune-related lncRNAs were further identified by comparing their expressions between bladder cancer and normal specimens^16,23^. With cyclically single pairing methods with 0-or-1 matrices, we identified immune-related lncRNA pairs. Combining univariate regression analyses and LASSO analyses, we developed a risk model for bladder cancer. Not using the median RS as the cutoff value that differentiated bladder cancer subjects into high- and low-risk subgroups, the AIC value of one-year survival was determined as the optimal cut-off value^16,24,25^. Furthermore, this risk model possessed distinct associations with survival status, age, stage and TNM of bladder cancer. According to multivariate regression analyses, the risk model might independently predict bladder cancer patients’ OS. In comparison to other clinical features, the risk model displayed the highest AUC of one-year OS, indicating that the model possessed the potential as a favorable predictor of bladder cancer. Moreover, this risk model was in relation to immune cell infiltrations and immune checkpoints. Intertumoral tumor-infiltrating immune cells may impact the responses to ICIs^26,27^. Here, by comprehensively utilizing XCELL, TIMER, QUANTISEQ, MCPCOUNTER, EPIC, CIBERSORT-ABS and CIBERSORT algorithms, we characterized the correlations between risk model and immune cell infiltrations. High-risk specimens possessed elevated infiltration levels of myeloid dendritic cells, B cells native, macrophages M0 and M2, neutrophils and T cells CD8 in bladder cancer. Also, GAL9 displayed elevated expression in low- than high-risk specimens while higher TIM-3 and PD1LG2 expressions were found in high-risk specimens than low-risk specimens. These data indicated that this risk model might be utilized for predicting immunotherapy response of bladder cancer.

Bladder cancer represents a complex malignancy correlated to high morbidity and mortality risks if not treated optimally. Neoadjuvant chemotherapy has been recommended prior to radical cystectomy for bladder cancer. Although the survival benefit is nearly 5–10%, some subjects cannot respond to chemotherapy^28^. Thus, identifying predictors may distinctly reduce side effects and miss the optimal time for surgery. Here, our data suggested that high-risk patients exhibited higher sensitivity to cisplatin in comparison to low-risk individuals. Inversely, subjects with low-risk were more sensitive to methotrexate than those with high-risk. Above data indicated that this risk model might possess the potential to predict the sensitivity to cisplatin and methotrexate for bladder cancer.

Due to high abundance, lncRNAs have distinct biological functions^29,30^. Our methods identified dysregulated immune-related lncRNAs and established the optimal immune-related lncRNA pairs. Hence, pairs with high or low expressions only were tested not detecting expression levels of each lncRNA. Our risk signature possessed the superiority in clinical practice for distinguishing high- and low-risk patients. Due to the closely correlations to immune-related genes, the selected lncRNAs potentially participated in modulating the immune microenvironment of bladder cancer. However, there are several limitations in our study. Firstly, because the lncRNA expression profiles of bladder cancer patients with complete survival time are not available in public databases, this study did not have an external validation to evaluate the performance of the prognostic immune-related lncRNA model. Therefore, more independent bladder cancer cohorts should be utilized for validating the risk model in our future studies. Furthermore, the functions of these lncRNAs and their interactions with immune-related genes will be confirmed based on in vitro and in vivo experiments.

Collectively, this prognostic signature constructed by 15 immune-related lncRNA pairs served as an independent predictor and displayed the favorable performance in predicting prognoses of bladder cancer. Also, it had the potential to predict immune landscape and chemotherapeutic response for bladder cancer patients.

## Materials and methods

### Data acquirement

Transcriptome profiles of bladder cancer (n = 414) and normal bladder specimens (n = 19) were retrieved from TCGA project (https://tcga-data.nci.nih.gov/tcga/). Through Ensembl (http://asia.ensembl.org), the Gencode (version 26) GTF file was obtained to annotate and differentiate mRNAs and lncRNAs^31^. Following removing specimens without clinical information or those with survival time of 0-day, 408 bladder cancer specimens and 19 normal bladder specimens were retained and complete clinical features of bladder cancer patients were listed in Supplementary table 5.

### Identifying immune-related lncRNAs

Totally, immune-related genes were obtained from the ImmPort database (http://www.immport.org). Supplementary table 6 listed the detailed information of these immune-related genes. Through Pearson correlation analyses, this study assessed the correlations between immune-related genes and extracted lncRNAs. The immune-related lncRNAs were screened according to correlation coefficient > 0.4 and p < 0.001.

### Identifying dysregulated immune-related lncRNAs

Differential expression analyses of the immune-related lncRNAs between tumor and normal bladder specimens were screened utilizing limma package^32^. The lncRNAs with |log fold-change|> 1.5 and false discovery rate (FDR) < 0.05 were screened. Above lncRNAs were visualized by heatmap package.

### Pairing dysregulated immune-related lncRNAs

Cyclically singly paring dysregulated immune-related lncRNAs were screened. The 0-or-1 matrices were developed if α = lncRNA-1 + lncRNA-2. α = 1 when lncRNA-1 expression was > lncRNA-2, while α = 0 when lncRNA-1 expression was < lncRNA-2. If the expression of lncRNA pair was 0 or 1, we thought there were no associations between this pair and prognoses, since the pair that did not a certain rank cannot be correctly predictive of patients’ prognoses. If the number of lncRNA pairs that expression was 0 or 1 occupied > 20% of entire pairs, this was an effective match.

### Establishing a prognostic risk model

Prognoses analyses of dysregulated immune-related lncRNAs were carried out through univariate cox regression models. LncRNAs with p < 0.05 could impact survival outcomes of bladder cancer. These lncRNAs were put into Least Absolute Shrinkage and Selector Operation (LASSO) model via glmnet package^33^. Penalty parameter tuning was carried out through ten‐fold cross‐verification. This analysis was run lasting 1000 cycles. The frequency of every pairing in the 1000-times-repeated LASSO model was retained and pairing with frequency > 100 times was chosen for constructing this model. Afterwards, a multivariate Cox regression model was conducted for determining the risk score (RS) through the coefficients and expressions of candidate lncRNA pairs according to the following formula: RS = $\mathop \sum \nolimits_{i = 1}^{k} \beta iSi$, where β represented the coefficient of lncRNA pair i and S represented the expression of lncRNA pair i.

### Evaluating the predictive efficacy of the prognostic risk model

Receiver operator characteristic (ROC) curves were depicted for assessing one-, three- and five-year overall survival (OS). By calculating the area under the curve (AUC), the predictive efficacy of the prognostic risk model was determined. The Akaike information criterion (AIC) value of each point for the one-year ROC curves was calculated for identifying the maximum inflection point, which was selected as the cut-off value for distinguishing patients into high and low risk subgroups. Survival status of each subgroup was visualized. Prognoses analyses of high and low risk subgroups were conducted through Kaplan–Meier curves. Differences in survival were determined with log-rank tests.

### Clinical feature assessment of the risk model

Associations between RS and clinical features (survival status, age, gender, grade, stage, T, N and M) were evaluated via chi-square tests. Also, RS was compared among different subgroups according to these clinical features. Univariate cox regression analyses were conducted for screening which factors could impact patients’ survival. Hazard ratio and p values were separately calculated. Utilizing multivariate cox regression analyses, indicators that were independently predictive of survival were determined. One-year ROC curves were plotted for comparing the predictive performance of risk model and other clinical features.

### Analysis of immune cell infiltrations

The known algorithms that included TIMER (version 2.0; http://timer.cistrome.org/)^34^, CIBERSORT (http://cibersort.stanford.edu/)^35^, XCELL (http://xCell.ucsf.edu/)^36^, QUANTISEQ (http://icbi.at/quantiseq)^37^, Microenvironment Cell Populations-counter (MCPcounter)^38^ and EPIC (http://epic.gfellerlab.org)^39^ were employed for inferring immune cell infiltrations of bladder cancer specimens on the basis of gene expression profiling. Spearman correlation analyses were carried out for estimating the associations between RS and immune cell infiltrations. Immune cell infiltrations between high- and low-risk subgroups were compared through Wilcoxon tests. Immune cells with p < 0.05 were screened and visualized into a lollipop diagram utilizing ggplot2 package.

### Associations between immune checkpoints and risk model

The expressions of immune checkpoints (CTLA4, LAG3, PDL1, PD1, TIGIT, GAL9, TIM-3 and PD1LG2) were quantified in every bladder cancer specimen. Their expressions were compared between high- and low-risk subgroups.

### Estimating the associations between chemosensitivity and risk model

The half inhibitory concentration (IC50) of chemotherapy drugs (cisplatin, methotrexate, vinblastine, gemcitabine and doxorubicin) was determined in every bladder cancer specimen utilizing pRRophetic package^40^. The differences in IC50 were compared between high- and low-risk subgroups.

### Statistical analyses

This study utilized R software (version 4.0.0: http://www.r-project.org) for conducting statistical analyses. The differences between two subgroups were estimated utilizing Wilcoxon rank sum tests. Meanwhile, three or more groups were compared through Kruskal–Wallis test. All statistical tests were two-sided when p < 0.05 indicated statistical significance.

## Supplementary Information

Supplementary Information 1.

Supplementary Information 2.

Supplementary Information 3.

Supplementary Information 4.

Supplementary Information 5.

Supplementary Information 6.

Supplementary Information 7.

Supplementary Information 8.

## Data Availability

All data generated or analyzed during this study are included in this article.

## Abbreviations

RNA-seq: RNA sequencing
TCGA: The Cancer Genome Atlas
lncRNAs: Long noncoding RNAs
FDR: False discovery rate
LASSO: Least Absolute Shrinkage and Selector Operation
RS: Risk score
ROC: Receiver operator characteristic
OS: Overall survival
AUC: Area under the curve
AIC: Akaike information criterion
MCPcounter: Microenvironment Cell Populations-counter
IC50: Half inhibitory concentration
