# Supplementary figures and images for: An immune-related lncRNA model for predicting prognosis, immune landscape and chemotherapeutic response in bladder cancer
Source: Sci Rep. 2022 Feb 25;12:3225. doi: 10.1038/s41598-022-07334-w (PMC8881497; doi:10.1038/s41598-022-07334-w)

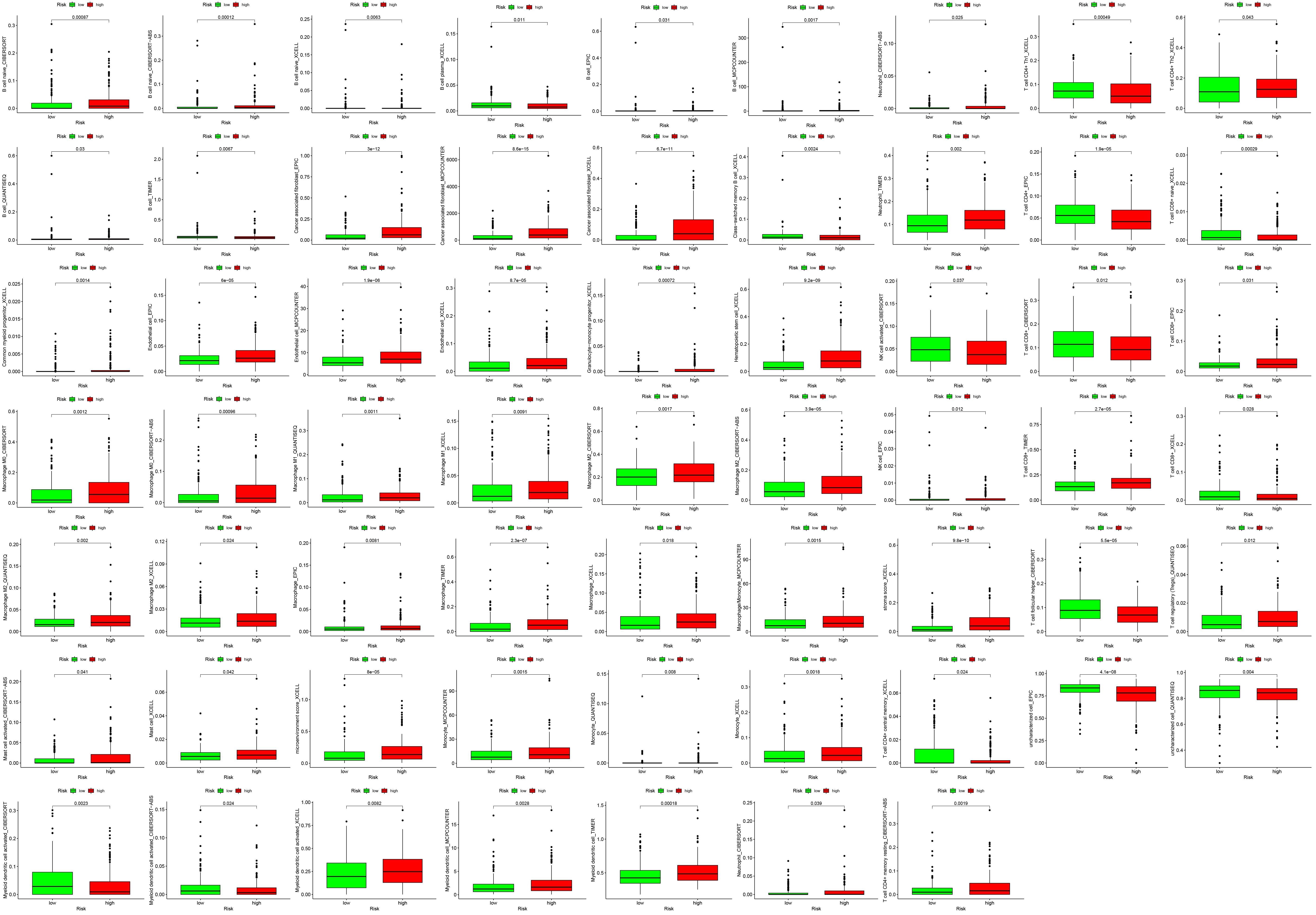

Supplement: Supplementary file 7 — Supplementary Information 7. [file 41598_2022_7334_MOESM7_ESM.tif]
