# Supplementary material for: An immune-related lncRNA model for predicting prognosis, immune landscape and chemotherapeutic response in bladder cancer
Source: Sci Rep. 2022 Feb 25;12:3225. doi: 10.1038/s41598-022-07334-w (PMC8881497; doi:10.1038/s41598-022-07334-w)
Supplement: Supplementary file 8 — Supplementary Information 8. [file 41598_2022_7334_MOESM8_ESM.docx]

**Additional files:**

Supplementary table 1. A list of immune-related lncRNAs.

Supplementary table 2. A list of differentially expressed immune-related lncRNAs in bladder cancer.

Supplementary table 3. Dysregulated immune-related lncRNA pairs in bladder cancer.

Supplementary table 4. Univariate cox regression analyses of immune-related lncRNA pairs that significantly affected bladder cancer prognoses.

Supplementary table 5. The detailed clinical information of bladder cancer patients.

Supplementary table 6. A list of immune-related genes.

Supplementary figure 1. Immune cell infiltrations in high- and low-risk bladder cancer specimens.
